# Attitudes toward mathematics and technology predict computational thinking more strongly than digital literacy in pre-service teachers

**DOI:** 10.3389/fpsyg.2026.1872825

**Published:** 2026-06-08

**Authors:** Katibe Gizem Yığ, Ayşe Gökçe-Ulutaş, Neşe Sevim-Çırak

**Affiliations:** Department of Elementary Mathematics Education, Mehmet Akif Ersoy University, Burdur, Türkiye

**Keywords:** attitudes toward mathematics and technology, computational thinking, digital literacy, pre-service teachers, teacher education

## Abstract

Teachers are expected to integrate technology effectively into instruction, which requires not only strong digital literacy but also well-developed computational thinking skills. In this context, this study examined the extent to which pre-service teachers’ computational thinking skills were predicted by demographic variables, digital literacy, and attitudes toward mathematics and technology. The study was conducted using a correlational design with 202 pre-service middle school mathematics teachers enrolled in a teacher education program at a public university. Hierarchical regression analysis was employed to determine the predictive contributions of the variables. In the initial model, gender and technology competence significantly predicted computational thinking skills, although the explained variance was relatively low. When digital literacy was added, the explained variance increased to 23.8%, and digital literacy emerged as a significant predictor, while demographic variables became non-significant. In the final model, attitudes toward mathematics and technology were included, increasing the explained variance to 47.7%. At this stage, attitudes toward mathematics and technology emerged as the strongest and only significant predictor. These findings suggest that affective factors may play a more central role than digital literacy and demographic characteristics in explaining computational thinking skills in teacher education contexts. The study contributes to the literature by providing an integrated perspective on cognitive and affective factors and offers implications for teacher education programs aiming to foster both technological competence and positive dispositions toward mathematics and technology.

## 1 Introduction

With the rapid advancement of digital technologies, their integration has become essential across various fields, including education. In fact, supporting individuals with the skills required in the digital era and fostering lifelong learning processes are among the primary objectives of contemporary educational policies (Angeli et al., 2016). Accordingly, the focus of educational research has shifted from the mere use of technology to its effective integration into teaching and learning processes. Within technology-enhanced learning environments, individuals are expected not only to utilize digital technologies but also to enhance higher-order thinking competencies, particularly problem-solving, analysis, and creative solution generation (National Research Council, 2012; Ministry of National Education [MoNE], 2023). Within this context, computational thinking (CT) has been increasingly recognized as a key construct in education.

CT is increasingly regarded as one of the essential 21st-century skills as it represents a comprehensive approach to problem-solving (Voogt et al., 2015). According to Wing (2006), CT encompasses skills such as analyzing complex problems, designing solution strategies, developing algorithms, and modeling data. It is not limited to the passive use of digital tools; instead, it empowers individuals to create novel tools and solutions with societal impact, and so it fosters innovation and creativity (Mishra et al., 2013; Yadav et al., 2014). For these reasons, CT has become a central goal in modern education.

The development of CT skills is not solely dependent on individual cognitive capacities; in fact, it is shaped by a range of contextual and pedagogical factors. Previous studies conducted at various educational levels, including with pre-service and in-service teachers, have explored the factors that contribute to the development of CT skills. Some of this research has examined the impact of different instructional approaches—such as digital games, project-based learning, and AI-supported learning tools—on students’ CT competencies, while others focus on factors that influence CT skills (Lin et al., 2024; Suparman et al., 2025). For example, Küçükaydın and Çite (2024) found that CT skills of the elementary students were significantly associated with several variables, such as the education level of their mother, students’ attitudes toward mathematics, and the integration of digital technologies into instructional practices. Similarly, Yıldız-Durak and Saritepeci (2018) identified thinking styles, mathematics achievement, and attitudes toward mathematics as significant predictors of CT skill levels of the middle school students. Likewise, Jiang et al. (2024) reported that STEM attitudes significantly predicted university students’ computational thinking skills, highlighting the importance of affective factors in CT development.

CT is also crucial for enabling individuals to develop systematic solutions to real-life problems (Wing, 2011). However, for children to acquire this skill from an early age, it is essential that teachers first possess the necessary competencies. Hence, the development of pre-service and in-service teachers in this field is critically important for fostering CT among K–12 students (Barr and Stephenson, 2011). In this regard, studies that investigate pre-service teachers’ current CT skill levels and address existing deficiencies are particularly valuable, as they hold the potential to influence the CT skills of future generations in their classrooms.

Despite this importance, research focusing on pre-service teachers’ CT skills remains relatively limited. Recent systematic review studies have indicated that further research is needed to better understand computational thinking in teacher education contexts and to examine how CT frameworks operate within teacher education settings (Listiaji and Molnár, 2025). Bower and Falkner (2015), for instance, found that many pre-service teachers demonstrate a limited and fragmented understanding of CT and struggle to articulate clear strategies for fostering these skills in the classroom. In a related study, Akgün (2020) reported a positive, but weak, correlation between CT and ICT competencies among pre-service teachers from various departments within a faculty of education. Furthermore, CT skills were reported to be positively associated with knowledge of computer hardware and software.

Among the various factors influencing CT, digital literacy (DL) has emerged as a particularly significant contributor. DL is defined as the ability to locate, evaluate, organize, and creatively use digital resources (Bawden, 2008). As Gilster (1997) famously stated, “DL is about mastering ideas, not keystrokes” (p. 1). DL is not limited to technical skills, such as tool use; it also includes critical thinking processes. DL encompasses the technical, cognitive, and socio-emotional skills necessary for effectively using digital technologies (Ng, 2012). In this sense, since DL supports the conscious use of information technologies, it is also considered to contribute to CT processes.

In addition to DL, attitude, particularly toward mathematics and technology, is considered another important factor associated with CT. Numerous studies conducted at the primary and secondary school levels have identified a significant relationship between students’ mathematical attitudes and their CT skills. For example, Küçükaydın and Çite (2024) demonstrated that even at the primary school level, students’ attitudes toward mathematics significantly affected their CT skills. At the middle school level, Alsancak-Sırakaya (2020) identified attitude toward math as a significant predictor of CT. Similarly, Yıldız-Durak and Saritepeci (2018) identified both mathematical achievement and attitude as significant predictors of students’ CT. These findings suggest that pre-service teachers’ attitudes toward mathematics and technology may also play an important role in developing their own CT skills.

While previous studies have primarily emphasized digital skills as key predictors of CT, comparatively fewer studies have examined the relative contribution of affective factors within the same analytical model. Addressing this gap, the present study investigates the unique and combined effects of DL and attitudes toward mathematics and technology on CT among pre-service mathematics teachers.

In light of the reasons mentioned above, this study aims to examine the extent to which pre-service middle school mathematics teachers’ CT skills are predicted by their DL levels and their attitudes toward mathematics and technology. In addition, demographic variables such as gender, age, and self-reported internet and technology skills were controlled to examine the unique contributions of the independent variables.

To be more specific, the research questions of this study are:

- To what extent do demographic variables (gender, age, and self-reported internet and technology competence) predict the CT skills of pre-service middle school mathematics teachers?
- To what extent does DL predict the CT skills of pre-service middle school mathematics teachers?
- To what extent do attitudes toward mathematics and technology predict the CT skills of pre-service middle school mathematics teachers?
- What are the unique contributions of DL and attitudes toward mathematics and technology to CT skills when controlling for demographic variables such as gender, age, and self-reported internet skills and technology competence?

## 2 Theoretical framework

The present study is theoretically grounded in Seymour Papert’s constructionism theory and Bandura’s Social Cognitive Theory. From a constructionist perspective, computational thinking develops through active engagement with technological tools, problem-solving activities, and knowledge construction processes (Papert, 1980). In this context, digital literacy can be considered a foundational competence that enables individuals to interact effectively with digital environments and computational tasks. Social Cognitive Theory further suggests that individuals’ beliefs, attitudes, and perceived competencies influence their learning behaviors and performance outcomes (Bandura, 1986). Accordingly, positive attitudes toward mathematics and technology may facilitate engagement in computational practices and contribute to the development of computational thinking skills among pre-service teachers.

### 2.1 Computational thinking

There is a broad consensus on the importance of CT, although its exact definition remains debated (Grover and Pea, 2013; Kalelioğlu et al., 2016). In the literature, computational thinking is framed as a way of approaching problems by drawing on ideas from computing to make sense of patterns, structures, and processes found in natural, social, and artificial systems (Csizmadia et al., 2015). The literature distinguishes two main perspectives on CT: domain-specific and domain-general (Tsai et al., 2021). From a domain-specific perspective, CT includes skills and knowledge required to solve problems in computer science or programming contexts, whereas the domain-general perspective emphasizes broader, transferable problem-solving skills applicable across everyday situations and disciplines (Tsai et al., 2021). CT also involves cognitive processes such as algorithm design, data representation, analysis, simulation, pattern recognition, generalization, and parallelization (Kang et al., 2023).

Wing (2006) conceptualizes CT as a way of thinking that applies computer science principles to problem solving and system design. She emphasizes that CT is not synonymous with programming, but rather involves thinking at multiple levels of abstraction and drawing on core concepts of computer science (Wing, 2006, 2008). From this perspective, CT models human problem-solving processes and integrates mathematical and engineering thinking in the analysis and design of systems, and is presented as a universally applicable form of analytical reasoning (Wing, 2008). Wing (2011) further refined CT as a thinking process involving the decomposition of complex problems, identification of essential details, and evaluation of outcomes to achieve efficient and effective solutions.

Building on this cognitive perspective, the International Society for Technology in Education (ISTE) and the Computer Science Teachers Association (CSTA) proposed an operational definition of CT. According to International Society for Technology in Education (ISTE) (2015), Computer Science Teachers Association (2011), computational thinking can be viewed as a structured way of approaching problems in which data are examined and represented through abstraction, solution strategies are developed using algorithmic reasoning, and problem-solving processes are extended and adapted to new situations. In this sense, CT enables individuals to formulate innovative questions and develop effective solutions through abstraction, decomposition, and systematic analysis of information (Kirçali and Özdener, 2023; Tang et al., 2020). From a computer science perspective, Aho (2012) defines CT as the analysis of problems in a way that allows them to be solved using algorithms and computational steps.

Synthesizing the literature, Selby and Woollard (2013) identified five fundamental mental strategies underlying CT across contexts: abstraction (focusing on essential details), decomposition (dividing problems into manageable parts), algorithmic thinking (applying step-by-step procedures), evaluation (assessing solution quality and efficiency), and generalization (applying patterns to new problems). Similarly, Korkmaz et al. (2017) conceptualized CT as encompassing critical thinking, creativity, collaboration, communication, algorithmic thinking, and problem solving, while Shute et al. (2017) defined CT through dimensions such as decomposition, abstraction, generalization, algorithmic design, debugging, and iteration. Despite these varying interpretations, prior reviews consistently indicate that abstraction, problem solving, algorithmic thinking, and automation are among the most frequently emphasized components of CT (Hsu et al., 2018; Kalelioğlu et al., 2016).

### 2.2 Computational thinking in education

The idea of CT has been discussed in the field of computer science since the 1960s (Grover and Pea, 2013). At first, it was regarded as an important skill for computer scientists, but over the past decade, there has been growing support for teaching CT in schools (Kong et al., 2019; Yadav et al., 2014). For instance, Wing (2006) argues that CT is a universal cognitive skill that can be applied to a wide range of problems in various domains, not just within the computer science domain. As a result, Wing (2006, 2011) redefined CT as an additional aspect of computer literacy and proposed its incorporation into school curricula. Similarly, Guzdial (2008) describes CT as a way of thinking that exceeds any single subject. Additionally, Benakli et al. (2017) and Wing (2014) claim that CT is an essential skill, similar to reading, writing, and basic arithmetic; therefore, it should be introduced at early stages of education. Today, there is general agreement among researchers that CT is a vital 21st-century skill that should be integrated across all levels of education, from early childhood through to university (Grover and Pea, 2013; Hsu et al., 2018; Shute et al., 2017; Snodgrass et al., 2016). Integrating CT into education not only boosts STEM learning but also helps bridge the gap between traditional classroom practices and real-world professional skills (Weintrop et al., 2016). According to the National Research Council (2011), there are several key benefits to teaching CT in educational settings. These include: (1) providing students new ways to explore and understand the world and scientific concepts; (2) automating repetitive tasks and managing complexity across learning environments; (3) enhancing capabilities in complex design and simulation; and (4) fostering personal expression and collaboration.

Barr and Stephenson (2011) emphasized that, when the role of computing in today’s world is considered, it is essential to introduce CT concepts to students much earlier, rather than waiting until they reach college. Similarly, Bower and Falkner (2015) argued that educational systems must go beyond teaching basic DL—such as familiarity with technological tools—and also focus on the CT processes necessary to comprehend the scientific principles behind technology.

### 2.3 Computational thinking and digital literacy

Gilster (1997) defines digital literacy (DL) as the ability to effectively and critically navigate, evaluate, and create information using various digital technologies. According to Yuan et al. (2021), it includes the ability to access, evaluate, and create digital content, which is essential for informed decision-making and active participation in a digital world. DL provides the foundational knowledge and skills necessary for students to engage with digital tools and information. Segredo et al. (2016) emphasize the need to move beyond simply teaching students the technical use of digital tools. They advocate for fostering adaptive skills that prepare learners to engage with future technological developments, especially in environments such as smart classrooms, and define CT as a key competency for supporting this adaptability. It also includes essential 21st-century skills such as problem-solving and creativity (Nouri et al., 2020). Thus, DL is closely associated with the development of skills such as computer programming and CT.

Gretter and Yadav (2016) argue that DL and CT are closely connected concepts that together support the development of digital proficiency. While DL provides the necessary skills to navigate, evaluate, and utilize digital environments effectively (Hague and Payton, 2010), CT provides the analytical frameworks and problem-solving methodologies that help users become digital creators rather than consumers (Brennan and Resnick, 2012). In fact, individuals with strong CT abilities can more effectively analyze digital information, understand algorithmic bias, and create meaningful digital solutions (Noble, 2018; Grover and Pea, 2013). Kafai and Burke (2014) emphasized that working with computational activities helps students better understand digital media and technology. At the same time, DL supports their ability to use computational tools effectively, indicating that DL and CT mutually reinforce one another. These skills enable students to either create digital content through programming or critically analyse such content using analytical reasoning. Gümüş et al. (2024) conducted research with 204 secondary school students, indicating that students’ DL positively influenced their confidence in computer programming and CT. The results also indicate that DL had an indirect effect on students’ confidence in CT. CT plays an important supportive role in DL, as it facilitates the development of students’ problem-solving and analytical skills (Law et al., 2008). It involves formulating problems in a way that a computer can assist in solving, which fosters logical thinking and systematic reasoning (Wing, 2006). Similarly, Yuliana et al. (2020) suggest that integrating CT into formal education is essential for enhancing DL. They emphasize that CT fosters both critical thinking and creativity by helping learners understand the underlying logic of digital processes. Individuals with this understanding are better prepared to use digital technologies in meaningful and informed ways. According to Gretter and Yadav (2016), DL and CT together represent a comprehensive range of 21st-century competencies—from creative production to critical reflection—that equip students to engage effectively in the digital age. To support this, teacher training programs need to prepare educators to teach both DL and CT. This involves learning teaching methods that effectively combine these skills across different subjects, creating a learning environment where students can build both their digital and computational abilities (Grover and Pea, 2013).

### 2.4 CT and attitudes toward mathematics

CT in mathematics education is not a recent development; it has long been a key component in the work of Papert (1980) with the Logo programming language. Currently, however, the curriculum appears to treat CT as a separate objective rather than integrating it into the teaching of existing subjects, as was done with Logo and mathematics. Nonetheless, “there is a natural (and historical) connection between CT and mathematics—in terms of logical structure and the ability to model mathematical relationships” (Gadanidis et al., 2017a, p. 77). Research suggests that integrating CT into mathematics can introduce alternative problem-solving approaches and broaden the range of mathematical topics accessible to students (Gadanidis et al., 2017b).

Studies indicate that mathematics is one of the key domains in which CT is actively applied (Benakli et al., 2017; Weintrop et al., 2016). CT components such as algorithmic thinking and abstraction strongly overlap with practices emphasized in mathematics education (Barr and Stephenson, 2011; Weintrop et al., 2016). Researchers such as Rich et al. (2019) and Weintrop et al. (2016) suggest that CT can be effectively incorporated into mathematics curricula. In particular, CT practices such as problem decomposition and algorithmic thinking provide a structured way of approaching complex mathematical tasks by breaking them into more manageable components (Weintrop et al., 2016).

Gonda et al. (2022) emphasize that using CT in education does not necessarily require technology. Instead, it involves applying robust problem-solving methods grounded in scientific reasoning, mathematical logic, and algorithmic thinking. Similarly, Kong et al. (2018) argue that teaching CT helps students solve problems creatively using technology. According to Gadanidis (2017), CT offers five main advantages in elementary math education: student empowerment, improved accessibility, simplified concepts, use of automation, and engagement with a real audience. Overall, the literature provides substantial evidence highlighting the close relationship between CT and mathematics education.

Sneider et al. (2014) emphasize that mathematical and CT overlap in core practices such as problem solving, modeling, and data analysis, particularly within science education contexts. Alsancak-Sırakaya (2020) demonstrated that positive attitudes toward technology-enhanced learning environments are associated with higher levels of CT. In contrast, Kang et al. (2023) developed a multidimensional assessment of CT and found that students studying mathematics and computer science demonstrated higher levels of CT than students from other disciplines, highlighting a strong connection between CT and mathematical skills.

Similarly, Benakli et al. (2017) suggest that engaging in hands-on computational activities with technology can provide rich contexts for developing students’ mathematical reasoning and problem-solving skills. Tomanova et al. (2024) found that students with limited math background can do well in decomposition and pattern recognition but often struggle with algorithmic thinking. In contrast, students with strong math skills perform well in all three areas, showing a close link between math ability and CT.

Barr and Stephenson (2011) further highlighted the importance of professional development for in-service teachers and the preparation of pre-service teachers to effectively implement CT in K-12 education. However, Bower and Falkner (2015) found that preservice teachers often lack a comprehensive understanding of CT and may not have clear strategies for incorporating it into their classrooms. In their study, preservice teachers also expressed the need to develop pedagogical skills related to CT, including understanding the curriculum, lesson planning, implementation strategies, and making connections to real-world examples. Likewise, Yadav et al. (2014) emphasized the importance of helping teachers understand CT in relation to the subjects they teach. Without this, teachers may only have a theoretical understanding of CT that is not applied effectively in the classroom.

## 3 Methods

### 3.1 Research design

This study aimed to examine the extent to which CT skills of pre-service middle school mathematics teachers are predicted by demographic variables (gender, age, and self-reported internet and technology skills), DL and attitudes toward mathematics and technology. Accordingly, a correlational research design was employed. A correlational research design is used to investigate the relationships between two or more variables (Tabachnick and Fidell, 2012). In this study, CT skills were treated as the dependent variable, while DL, attitudes toward mathematics and technology were treated as independent variables. Variables identified as being associated with CT skills—namely gender, age, internet usage skills, and technology usage skills—were included as control variables.

### 3.2 Participants of the study

This study involved 202 pre-service middle school mathematics teachers from the Department of Mathematics Education of a public university located in southern Türkiye. Of the participants, 72.3% were female (*N* = 146) and 27.7% were male (*N* = 56). The demographics of the participants are presented in Table 1 in detail.

**Table 1:** The demographics of the participants.

Demographics	Variables	*N*	*F*
Gender	Female	146	72.3
	Male	56	27.7
Class	Freshman	56	27.7
	Sophomore	50	24.8
	Junior	32	15.8
	Senior	64	31.7
Internet Skills	Low	7	3.5
	Intermediate	113	55.9
	High	82	40.6
Technological Competence	Low	19	9.4
	Intermediate	128	63.4
	High	55	27.2
Age	18	18	8.9
	19	29	14.4
	20	42	20.8
	21	44	21.8
	22	30	14.9
	23	23	11.4
	24+	16	8
Total		202	100.0

### 3.3 Ethical approval and consent to participate

This study was approved by the Ethics Committee of [Institution name omitted for blind review] (Meeting Date: May 7, 2024; Meeting No: 2024/05; Decision No: GO 2024/293). All procedures performed in this study were conducted in accordance with the ethical standards of the institutional ethics committee and national ethical guidelines. Participation in the study was voluntary, and informed consent was obtained from all participants.

### 3.4 Instruments

#### 3.4.1 Computational thinking skills scale (CTS)

In this study, the CTS developed by Korkmaz et al. (2017) was used to assess participants’ CT skills. The scale consists of 29 items. Example items from the CTS “I trust my intuitions and feelings of “trueness” and “wrongness” when I approach the solution of a problem,” “I like solving problems related to group project together with my friends in cooperative learning”, and “I cannot produce so many options while thinking of the possible solution ways regarding a problem”. Responses were rated on a 5-point Likert scale (1 = never, 2 = rarely, 3 = sometimes, 4 = generally, and 5 = always) and comprises five factors: creativity (8 items), algorithmic thinking (6 items), cooperativity (4 items), critical thinking (5 items), and problem-solving (6 items). The researchers calculated the Cronbach’s alpha coefficient for the overall scale as 0.81 and for this study, the Cronbach’s alpha coefficient for the overall scale was calculated as 0.85.

#### 3.4.2 Mathematics and technology attitude scale (MTAS)

The mathematics and technology attitude scale (MTAS) was developed by Pierce et al. (2007) and adapted into Turkish by Çalışkan Dedeoğlu et al. (2021). The scale includes 20 items. Sample items include “If I make mistakes, I work until I have corrected them”, “I can master any computer program needed for school”, and “I am interested to learn new things in mathematics”. Responses were rated on a 5-point Likert scale (1 = never, 2 = rarely, 3 = sometimes, 4 = generally, and 5 = always) across 5 factors: Mathematics Confidence (4 items), Confidence with Technology (4 items), Attitude toward Learning Mathematics with Technology (4 items), Behavioral Engagement (4 items), and Affective Engagement (4 items). For each factor, a minimum of 4 and a maximum of 20 points can be obtained. Scores of 17 and above indicate a high level of attitude, scores between 13 and 16 indicate a moderate attitude, and scores of 12 and below suggest a negative attitude or a neutral stance toward the scale. Çalışkan Dedeoğlu et al. (2021) calculated the Cronbach’s alpha coefficient for the entire scale as 0.82. For this study, the Cronbach’s alpha coefficient for the entire scale was calculated as 0.83.

#### 3.4.3 Digital literacy scale

The digital literacy scale was developed by Ng (2012) and adapted into Turkish by Üstündağ et al. (2017). The scale consists of 10 items. Sample items from the DL scale include: “I frequently obtain help with my university work from my friends over the Internet e.g. through Skype, Facebook, Blogs” and “I have the technical skills I need to use ICT for learning and to create artifacts (e.g. presentations, digital stories, wikis, blogs) that demonstrate my understanding of what I have learnt”. Responses were rated on a 5-point Likert scale (1 = strongly disagree, 2 = disagree, 3 = neutral, 4 = agree, and 5 = strongly agree) and comprises a single factor. Üstündağ et al. (2017) reported a Cronbach’s alpha coefficient of 0.86 for the overall scale, while in the present study, the Cronbach’s alpha coefficient was calculated as 0.87.

### 3.5 Data analysis

In this study, data were examined by using hierarchical multiple regression analysis. All statistical analyses were conducted using IBM SPSS Statistics 25. Prior to the analysis, the assumptions of multiple linear regression required for hierarchical modeling were tested. For hierarchical multiple regression, the dependent variable should be continuous, and the residuals should be approximately normally distributed (Tabachnick and Fidell, 2012). Gender, internet skills, and technology competence variables were categorical variables; therefore, dummy variables were created for these variables. Gender was coded as female = 1 and male = 0, whereas internet skills and technology competence were recoded into binary variables (high/medium = 1, low = 0). The resulting dummy variables were entered into the hierarchical multiple regression model. In addition to normality, multicollinearity was also evaluated. Correlation coefficients between variables should be below 0.80, VIF values should not exceed 10, and tolerance values should be greater than 0.10 (Tabachnick and Fidell, 2012). In this study, VIF values ranged from 1.003 to 1.798, tolerance values ranged from 0.328 to 0.394, and correlation coefficients ranged from −0.319 to 0.687, indicating no multicollinearity concerns. Finally, to assess autocorrelation, the Durbin–Watson statistic was calculated (2.20), indicating that the residuals did not exhibit autocorrelation. Hierarchical regression analysis employs a block-wise approach in which different groups of variables are entered sequentially to control for their effects. Accordingly, the analysis was conducted in three steps. At each step, the effects of previously entered variables were controlled, allowing the unique contribution of the newly added variable group to be examined. The order of the variables included in the model was determined based on the literature review. Demographic variables (gender, age, internet skills, and technology competence) were controlled for because prior research suggests that these variables may influence pre-service teachers’ computational thinking skills. In Step 1, gender, internet skills, technology competence (converted into dummy variables), and age were included in the model. In Step 2, DL was added, and in Step 3, attitudes toward mathematics and technology were incorporated into the model.

## 4 Findings

### 4.1 Descriptive analysis

The descriptive analysis results regarding DL, attitudes toward mathematics and technology, and CT skills are presented in Table 2.

**Table 2:** Descriptive statistics on DL, mathematics and technology attitude, CT skills of the participants.

Variables	Number of items (k)	Min score	Max score	Mean	Mean/k	Std. deviation
TDL	10	15	50	34.35	3.44	7.22
TAMT	20	11	20	79.00	3.95	7.71
MC	4	8	20	17.02	4.26	2.20
ALMT	4	5	20	15.70	3.93	2.58
CTU	4	8	20	14.36	3.59	3.18
AE	4	4	20	17.45	4.36	2.13
BE	4	54	98	14.47	3.62	1.76
MTAS-T	29	14	30	112.78	3.89	11.79
AT	6	4	43	22.94	3.82	3.42
COL	4	6	30	14.52	3.63	4.04
PS	6	11	25	24.38	4.06	4.04
CTH	4	21	40	18.30	4.58	3.12
CR	8	86	145	32.64	4.08	3.34

TDL, Total digital literacy; TAMT, Attitudes toward mathematics and technology; MC, Mathematics confidence; ALMT, Attitude toward learning mathematics with technology; CTU, Confidence in technology use; AE, Affective engagement; BE, Behavioral engagement; MTAS-T, Total score of the mathematics and technology attitude scale; AT, Algorithmic thinking; COL, Collaboration; PS, Problem solving; CTH, Critical thinking; CR, Creativity.

The findings indicate that the participants’ DL levels were moderate. According to the analysis results, the participants’ attitudes toward mathematics and technology were also at an average level. Additionally, the relatively large standard deviation values across the variables indicate considerable variability in participants’ responses, suggesting differences in their levels of computational thinking, digital literacy, and attitudes toward mathematics and technology. Within the subdimensions, the highest mean scores were observed for Mathematics Confidence (M = 4.36, SD = 2.13) and Affective Engagement (M = 4.26, SD = 2.20), whereas the lowest mean score was observed for Confidence in Technology Use (M = 3.59, SD = 3.18). Additionally, the participants’ CT skills were found to be at a moderate level. Regarding CT skills, the highest mean score was observed in Critical Thinking (M = 4.58, SD = 3.12), while the lowest mean score was observed in Collaboration (M = 3.63, SD = 4.04) (Table 2).

According to the results of the Pearson correlation analysis, a positive and strong significant relationship was found between attitudes toward mathematics and technology and CT (*r* = 0.687, *p* < 0.01). A positive and moderate significant relationship was also identified between attitudes toward mathematics and technology and DL (*r* = 0.643, *p* < 0.01). Furthermore, a positive and moderate significant relationship was found between CT and DL (*r* = 0.460, *p* < 0.01) (Table 3).

**Table 3:** Correlations among the study variables.

Variables	1	2	3
1. Attitudes toward mathematics and technology	—		
2. Computational thinking (CT)	0.687**	—	
3. Digital literacy (DL)	0.643**	0.460**	—

*N* = 202, **p < 0.01.

## 5 Findings related to hierarchical regression analysis

Hierarchical regression analysis was conducted to examine the predictive roles of DL and attitudes toward mathematics and technology on pre-service middle school mathematics teachers’ CT skills. The study employed a three-step hierarchical approach, with predictors entered in the following order: demographic variables, DL, and attitudes toward mathematics and technology.

The first step of the regression analysis included only demographic variables as a predictor (Step 1). This model significantly predicted CT skills of the participants (*R* = 0.321, *R*^2^ = 0.103, *F*(4, 197) = 5.66, *p* < 0.001). Gender was found to be a significant predictor (*β* = −0.147, *p* < 0.05), as was technology competence (*β* = 0.178, *p* < 0.05). Overall, demographic variables predicted approximately 10% of the total variance. In the second step, the DL level was added to the model (Step 2). The model continued to significantly predict participants’ CT skills (*R* = 0.488, *R*^2^ = 0.238, *F*(5, 196) = 12.26, *p* < 0.001). DL level emerged as a significant predictor (*β* = 0.398, *p* < 0.001), and the model explained approximately 24% of the total variance.

The final step involved the addition of Mathematics and Technology Attitude level (Step 3). The overall model continued to predict participants’ CT skills (*R* = 0.690, *R*^2^ = 0.477, *F*(6, 195) = 29.60, *p* < 0.001), explaining nearly 48% of the total variance. In this model, DL was no longer a significant predictor (*β* = 0.019, *p* > 0.05), whereas attitude toward mathematics and technology showed a significant positive association with CT skills (*β* = 0.655, <0.001). Although the increase in R2 across models was significant, attitudes toward mathematics and technology added in Step 3 (*R* = 0.690, *R*^2^ = 0.477, *p* < 0.001) emerged as the most significant predictor of CT skills of pre-service middle school mathematics teachers (Table 4).

**Table 4:** Summary of hierarchical regression models predicting CT skills.

Variables		β	*t*	*p*
1	(Constant)		13.971	0.000
	Gender	−0.147	−2.162	0.032
	Internet skills	0.082	1.053	0.294
	Technology competence	0.178	2.267	0.025
	Age	0.133	1.968	0.050
	(*R* = 0.321, *R*^2^ = 0.103, *F*(4, 197) = 5.66, *p* = 0.00)			
2	(Constant)		11.222	0.000
	Gender	−0.083	−1.299	0.195
	Internet skills	0.067	0.924	0.357
	Technology competence	0.070	0.928	0.354
	Age	0.082	1.302	0.195
	Digital literacy	0.398	5.897	0.000
	*R* = 0.488, *R*^2^ = 0.238, *F*(5, 196) = 12.26, *p* = 0.00			
3	(Constant)		4.021	0.000
	Gender	−0.029	−0.543	0.588
	Internet skills	0.056	0.941	0.348
	Technology competence	0.012	0.193	0.847
	Age	0.002	0.040	0.968
	Digital literacy	0.019	0.281	0.779
	Attitudes toward mathematics and technology	0.655	9.424	0.000
	*R* = 0.690, *R*^2^ = 0.477, *F*(6, 195) = 29.60, *p* = 0.00			

Gender was dummy coded (0 = male, 1 = female).

## 6 Discussion

One of the most notable findings of this study is that attitudes toward mathematics and technology emerged as the strongest predictor of CT when all variables were considered simultaneously.

Although demographic variables and DL played a role in earlier stages of the model, the final results indicate that attitudes toward mathematics and technology have a more decisive influence on CT skills. This result indicates that affective and attitudinal factors may play a more central role than technical competencies alone in the development of CT skills.

In the first model, gender and technology competence were found to be significant predictors of CT, although the overall explained variance of the model remained relatively low. Specifically, 10.3% of the variance was explained by gender and technology competence, whereas internet skills and age did not have a significant effect on CT. This finding is consistent with the previous studies conducted in the literature. For example, Wing (2006) argues that CT emphasizes abstraction, problem decomposition, algorithmic reasoning, and analytical thinking. As stated by Mishra et al. (2013) and Yadav et al. (2014), CT is not about the passive use of digital tools; instead, it helps individuals to use the digital tools creatively. Similarly, Bower and Falkner (2015) noted that familiarity with digital tools alone does not necessarily translate into higher-order problem-solving abilities which is also indicated in this study.

In the second model, DL skills were added, and it is seen that 23.8% of the variance is explained by DL. In this model, DL emerged as a strong and statistically significant predictor of CT while gender and technology competence lost their significant effects on CT. This finding indicates that DL contributed to the prediction of CT in the intermediate model, although its effect became non-significant in the final model. The findings of this study are consistent with the studies that investigate the relationship between DL and CT. For instance, Gretter and Yadav (2016) conceptualize DL and CT as complementary components of 21st-century skills. Likewise, Kafai and Burke (2014) emphasize that engagement in computational activities enhances individuals’ understanding of digital media and supports meaningful participation in digital cultures. In a middle school context, DL was shown to be a significant determinant of students’ self-efficacy in both computer programming and CT (Gümüş et al., 2024). George-Reyes et al. (2021) examined the relationship between DL and CT and emphasized that these concepts have an interwoven relationship. Similarly, Gretter and Yadav (2016) indicated that DL and CT are related components of 21st-century skills, while Yuliana et al. (2020) pointed out that the integration of CT into formal education strengthens DL. The findings of this study provide empirical evidence that digital literacy was a significant predictor of computational thinking in the intermediate model; however, its effect became non-significant in the final model. Therefore, based on the relationship between DL and CT, and the results of this study, it can be suggested that the pedagogical and cognitive development of pre-service teachers should be supported together.

In the final stage, attitudes toward mathematics and technology were added to the model, and the explained variance increased from 23.8 to 47.7% which indicates that attitudes toward mathematics and technology have a critical role in predicting the CT skills of pre-service mathematics teachers. However, DL became non-significant in the final model. This finding suggests that the predictive power of DL may be reduced when affective variables such as attitudes toward mathematics and technology are taken into account. Similar patterns have been reported in the literature, where DL was found to influence CT both directly and indirectly through related constructs such as programming self-efficacy (Gümüş et al., 2024). One possible explanation for the loss of significance of digital literacy in the final model is that positive attitudes toward mathematics and technology may influence how pre-service teachers use their digital competencies to support computational thinking skills. Although digital literacy provides the technical and cognitive skills necessary to engage with digital environments, positive attitudes may increase individuals’ willingness, motivation, and confidence to use these skills for computational thinking. In this sense, affective factors may play a more central role in supporting the development of CT skills. This interpretation is also consistent with Social Cognitive Theory, which emphasizes the influence of beliefs, attitudes, and perceived competencies on learning behaviors and performance outcomes (Bandura, 1986). However, further studies employing mediation analyses are needed to examine the potential indirect pathways in the present context.

In the final model, attitudes toward mathematics and technology emerged as the only and the strongest predictor of CT skills. The relatively high level of explained variance obtained in this model should be interpreted in relation to the research context, as explained variance values in educational research are typically evaluated contextually rather than based on fixed thresholds (Henson and Roberts, 2006). Early work by Papert (1980) using the Logo programming language highlighted the close connection between CT and mathematics, demonstrating the potential of computational environments to support mathematical reasoning and problem-solving. CT and mathematical thinking include similar cognitive processes such as abstraction, logical reasoning, and algorithmic problem solving (Gadanidis et al., 2017; Benakli et al., 2017). Similarly, studies have also shown that mathematics is one of the primary domains in which CT is naturally applied (Weintrop et al., 2016). In fact, the studies in the literature suggest that positive attitudes toward mathematics are closely related to students’ CT skills (e.g., Alsancak-Sırakaya, 2020; Tomanova et al., 2024). Since mathematical thinking has similarities with algorithmic reasoning and problem-solving processes (Weintrop et al., 2016; Gadanidis et al., 2017), it can be argued that positive attitudes toward mathematics and technology provide a supportive cognitive and affective foundation for CT. For instance, Alsancak-Sırakaya (2020) found that positive attitudes toward mathematics significantly predict higher levels of CT. Similarly, Kang et al. (2023) reported that students from mathematically intensive disciplines demonstrated higher performance in CT, including abstraction, decomposition, and algorithmic thinking. Tomanova et al. (2024) further showed that while students with limited mathematical backgrounds may succeed in basic pattern recognition and decomposition, they often struggle with algorithmic thinking, which indicates the importance of a mathematical background. The findings highlight the need to support not only technical and cognitive skills, such as DL, but also affective dimensions, including attitudes toward mathematics and technology, in fostering the development of CT skills.

To sum up, this study shows that the influence of demographic variables (gender, technology competence) on CT skills is limited, and these effects are better explained through cognitive and affective skills, such as DL and attitudes toward mathematics and technology. In the literature, it is suggested that the focus should be on encouraging deep thinking, mathematical reasoning, and positive attitudes toward solving problems with technology rather than just using tools for CT (Barr and Stephenson, 2011; Yadav et al., 2014). Therefore, when considering teacher training, it can be said that deficiencies arising from differences in gender and technological competence may be addressed through educational interventions that support these affective and cognitive characteristics.

## 7 Conclusion

This study was conducted in order to examine the relationships between pre-service mathematics teachers’ CT, DL, and attitudes toward mathematics and technology by using hierarchical regression analysis. The findings indicate that attitudes toward mathematics and technology were the strongest predictors of CT, whereas the predictive effect of DL became non-significant in the final model. While gender and technology competence showed limited predictive power at first, they lost their effect when DL and attitudes toward mathematics and technology were introduced into the models.

This study suggests that DL may support CT since it helps people analyze information, think critically, and interact effectively with digital tools. However, the strongest predictor of CT was attitudes toward using technology in mathematics. This suggests that CT is closely tied to mathematical thinking and problem-solving. Feeling confident and developing positive attitudes about using technology in math play a key role in developing these skills.

## 8 Limitations

This study includes several limitations. First, the study relied on self-reported data, which may be subject to response bias and may not fully capture participants’ actual CT abilities, DL levels, or technology-related competencies. Future research could incorporate performance-based assessments or mixed-method approaches to provide a more comprehensive understanding of CT.

Second, the data were obtained from 202 students from the Department of Mathematics Education of a public university located in southern Türkiye, which limits the ability to draw causal conclusions. Furthermore, the sample shows a gender imbalance, with females representing 72.3% of the participants. Although this may limit the generalizability of the findings across gender groups, this distribution is consistent with the demographic structure of elementary mathematics teacher education programs in Türkiye. According to the Council of Higher Education [YÖK] (2026), 74.6% of the students enrolled in elementary mathematics teacher education programs are female, whereas 25.4% are male. In other words, the results are specific to the participant group included in this study, and differences in educational level, cultural context, or disciplinary background may yield different outcomes. Conducting similar studies with more diverse samples would strengthen the robustness of the conclusions.

## 9 Implications

### 9.1 Educational implications

The findings of this study have important implications for educational practice. The strong predictive role of attitudes toward mathematics and technology suggests that CT instruction could be closely integrated with mathematics education and emphasize algorithmic reasoning, abstraction, and problem-solving. Educators may design learning environments that promote positive attitudes toward mathematical thinking supported by technology.

Additionally, the significance of DL highlights the need for instructional approaches that go beyond basic tool use and focus on critical evaluation, information management, and creative digital production. Integrating DL with CT activities can help learners become not only consumers of technology but also analytical and reflective problem solvers.

### 9.2 Implications for teacher education

The results also underscore the importance of teacher preparation and professional development. Teachers need support in understanding CT as a cognitive and mathematical practice rather than a purely technical skill. Teacher education programs should provide opportunities for preservice and in-service teachers to develop pedagogical strategies that integrate CT, DL, and mathematics within and across disciplinary contexts.

### 9.3 Research implications

For future research, these findings suggest the need to explore the relationships between DL, attitudes toward mathematics and technology, and CT using longitudinal designs, experimental interventions, and advanced analytical approaches such as structural equation modeling (SEM). Such approaches may provide deeper insights into how CT develops and how these relationships evolve over time. Additionally, as the present study did not include any instructional interventions, future experimental studies could be designed to examine how targeted interventions—particularly those aimed at improving attitudes toward mathematics and technology—may influence the development of CT skills.

## Data Availability

The raw data supporting the conclusions of this article will be made available by the authors, without undue reservation.
